# Neurofeedback on twitter: Evaluation of the scientific credibility and communication about the technique

**DOI:** 10.1016/j.heliyon.2023.e18931

**Published:** 2023-08-07

**Authors:** Silvia Erika Kober, Finn Buchrieser, Guilherme Wood

**Affiliations:** Department of Psychology, University of Graz, Austria

**Keywords:** Neurofeedback, Twitter, Scientific communication, Alternatve medicine, Neuroenhancement

## Abstract

Neurofeedback is a popular technique to induce neuroplasticity with a controversial reputation. The public discourse on neurofeedback, as a therapeutic and neuroenhancement technique, encompasses scientific communication, therapeutic expectations and outcomes, as well as complementary and alternative practices. We investigated twitter publications from 2010 to 2022 on the keyword “neurofeedback”. A total of over 138 k tweets were obtained, which originated from over 42 k different users. The communication flow in the neurofeedback community is mainly unidirectional and non-interactive. Analysis of hashtags revealed application fields, therapy provider and neuroenhancement to be the most popular contents in neurofeedback communication. A group of 1221 productive users was identified, in which clinicians, entrepreneurs, broadcasters, and scientists contribute. We identified reactions to critical publications in the twitter traffic and an increase in the number of tweets by academic users which suggest an increase in the interest on the scientific credibility of neurofeedback. More intense scientific communication on neurofeedback in twitter may contribute to promote a more realistic view on challenges and advances regarding good scientific practice of neurofeedback.

## 1 Introduction

Neurofeedback is a popular technique to train the self-regulation of brain activity [1]. Neurofeedback is largely employed for diverse purposes, which range from motor rehabilitation, psychiatric disorders [2], attention disorders and hyperactivity [3], to epilepsy [4], insomnia, menopause, pre-menstrual tension, migraine, etc. [5]. However, for most these applications clinical evidence of efficacy is lacking completely or is at least limited [6]. In a series of critical papers [[6], [7], [8]], showed how low is the interest in key mechanisms of neurofeedback [8] such as psychosocial effects [9]. In particular [8], discuss a hitherto rather insufficient contribution of neurofeedback providers and specialty associations to promote scientific and evidence-based clinical neurofeedback. Do these particularities of neurofeedback influence the public discourse on the technique?

In the present study, the public discourse on neurofeedback will be examined using the Twitter platform. Twitter is a microblogging platform with over 350 million users worldwide and an important communication channel among scientists, in science-to-public communication, peer-to-peer interactions [10], health information seeking [11] as well as scientific journalism [12,13]. Twitter is also a channel of communication between companies, clinicians, and their customers/clients as they can announce their products and services, gather feedback on them and thereby construct a reputation [14]. As a popular technique to influence brain activity, taught and certified by several specialty associations world-wide [15], neurofeedback enjoys a considerable presence on Twitter and ascertains the existence of material for the present analysis.

When studying the way neurofeedback providers present their services to the public in their websites [16], concluded for a substantial divergence between evidence and the marketing promises of neurofeedback services. These authors recently investigated claims made by US neurofeedback providers in their websites and observed that only 36% of providers had a relevant medical or psychological degree while approximately 75% employed language characterized by the authors as related to complementary or alternative medicine. These findings are in line with the gap between the commercial exploitation of neurofeedback and an evidence-based form of treatment, as pointed out by Ref. [8]. [17] examined the Twitter presence of providers of treatments lacking scientific evidence and observed the lack of a stable community of promoters and supporters. These authors identified the presence of transient collectives rather than long-term identifiable groups. Therefore, an analysis of the level of activity in individual profiles is also relevant to characterize the communication patterns related to neurofeedback.

No data is available on how clients look for information on neurofeedback treatment or peer-to-peer interactions on social media. A review on health information seeking in social media [11] suggests that social media are consulted in search for peer-to-peer interactions and social and emotional support. Moreover, the motives of patients and health professionals to use social media in health care differ considerably [18]. These authors found out that patients use Twitter to increase knowledge and exchange advice and Facebook for social support and exchanging advice, while professionals use LinkedIn and Twitter for communication with colleagues and marketing.

Relevant events determine the number and contents of twitter publications [19,20,21]. To evaluate the impact of scientific progress on the public opinion about neurofeedback, we selected the time point of a few scientific and non-scientific events and measured the reaction of the public discourse on neurofeedback. For instance, the case of Neurocore in the US also brought Neurofeedback to the media and generated a reaction in twitter [22,23]. Moreover, critical papers by Refs. [24,7,6] expressed several concerns regarding the quality and quantity of clinical evidence of neurofeedback efficacy, among them the necessity to perform more placebo-controlled studies. Did these papers change the awareness for good scientific practices around neurofeedback in the twitter network? If the message of these papers reaches scientists, some of these concerns should be detectable in their social media as well. However, given the exaggerated propensity to believe in techniques connected to brain activity [25,26] and the optimism regarding their capabilities and soundness [27,28], the impact of critical literature may remain modest. For this reason, we will examine the occurrence of keywords connected to the topic of randomized controlled trials (RCTs), placebo, evidence-based treatment, etc. Moreover, an international consensus paper on quality criteria for neurofeedback studies was published in 2020, which may also have generated an impact on social media [29]. This best practice checklist was already published in 2019 as a preprint on PsyArXiv [30]. Typically, after a burst of interest in a certain topic, changes in vocabulary can be detected [19]. In the case of neurofeedback, we expect that the thematic of RCT, replicability, reproducibility, and open science becomes more present after the above-mentioned publications.

### 1.1 Research questions

First, we are interested in the question: who tweets about neurofeedback? As the most popular social media used for science communication, Twitter provides material to understand the public communication on the topic of neurofeedback. One of the aims of the present study is to provide a tentative characterization of user profiles communicating on the topic neurofeedback. Twitter profile descriptions consist of a 160-character short description of the user, are considered an expression of aspects of their social identity [31] and can help to understand users' interests on and motivations for interaction with Twitter contents. In combination with other metadata, Twitter short bios convey information on several user's characteristics [20] as well as mental status [32]. After identifying user groups, we analyze publication activity among the different profile categories as well as the number of followers.

Second, we address the question of which contents are related with neurofeedback in Twitter interactions. Hashtags are used to index and categorize content [33] and can be seen as distilled versions of users’ conceptual frameworks and viewpoints associated with specific topics [34]. Scientific communication on twitter is characterized by dissemination much more than by interaction [35]. Most tweets [22,23] on scientific communication only contain a link and are retweeted without original contribution [36]. For this reason, a specific analysis of the hashtags in each tweet as well as the user profile descriptions can be particularly informative. More importantly, hashtags define communities and communication spaces for users to represent themselves and identify with other users [37].

Finally, we are also dealing with the questions: Do scientific and critical findings on neurofeedback generate an impact on neurofeedback-related Twitter traffic? Do scientific advancements/criticism achieve the public in a substantive way as a digital advocacy tool [38] or is the communication on neurofeedback on twitter rather indifferent to it? The effectiveness of neurofeedback has been heavily criticized in the scientific literature but it is unclear to which extent the public is aware of these developments. The observation of the impact of concerns about the scientific credibility of neurofeedback research on the twitter community can be revealing about the permeability to scientific discourse as well as the effectiveness of science-to-public communication.

## 2 Methods

Tweets published between January 01, 2010 and May 31, 2022 were harvested using the twitter API v.2.0 through the r package academictwitteR [39]. Individual profiles were harvested using the API v.1.0 through the package twitteR [40]. All tweets analyzed in this study were obtained with the query “*neurofeedback*”. These tweets were searched for hashtags and the number of followers and following profiles of each author. To answer the first research question, a group of productive users was defined operationally as those with at least 10 original tweet publications containing the keyword “neurofeedback” in the time interval indicated above (see the next section for further details). Based on the author_id of each tweet, the profile description of these users (i.e. short bio) was obtained. When tweets are posted in response to a tweet (known as a reply), or in response to a reply, a conversation_id is available so that interactions between users can be measured and analyzed. Conversation_ids were also obtained to establish the frequency and depth of interaction between users posting on neurofeedback. The date a conversation began was used to localize conversations in time.

### 2.1 Profile analysis

Profile descriptions (“twitter bios”) contain 160 characters long personalized text. Profile description was obtained for each twitter profile with at least 10 posts on neurofeedback published in the time interval defined for data collection. A total of 1221 profiles fell into this category, 90 of them had a blank profile description and were disconsidered in further profile analyses. The function sentiment. match (.) of the package *sentiment. ai* [41] was employed to classify the profiles by means of category matching (model = “en.large”, method = “conda”, python version 3.7.4). Sentiment. ai is a text embedding deep learning engine with multiple functions including category matching given a semantic space consisting of collections of attributes. Collections of attributes were built based on informal inspection of short bio descriptions and employed as a semantic reference for the process of category matching. The terms employed for category classification designated groups of users depending on their interests on neurofeedback being scientific (“institution”, “scientist”, “academic”, “professor”, “student”, “researcher”, “university”, “research center”), broadcasting (“journalist”, “journal”, “radio”, “bot”, “podcast”, “conference”), entrepreneurship (“entrepreneur”, “sport”, “company”, “coach”, “CEO”), or clinical utility (“therapist”, “clinician”, “practitioner”). Selection of terms was performed by one of the authors after examining the contents of a couple of hundreds of profile descriptions (G.W.). The cosine similarity [42] of the profile description with the most similar attribute from our list was calculated and thresholded at the minimum value 0.15. Profiles with values lower than that (i.e. 341 profiles) were disconsidered in further analyses. The remaining profiles were categorized either as scientist (n = 164), broadcast (n = 67), entrepreneur (n = 76) or as clinician (n = 481). To validate the classification of profiles in the categories named above, we computed the number of followers and the number of profiles followed by each individual. We expect that broadcast profiles to have a larger number of followers and also to follow the most different profiles, since a large network is mandatory for their broad purpose. We also expect entrepreneurs to have a large number of followers, since companies invest in advertisement and have national as well as international costumers. We expect practitioners to have more circumscribed numbers of followers and also to follow not too many profiles, since their practical scope of action is necessarily more locally limited. Scientists posting on twitter usually have many other scientists as their followers [35], however, it is less clear how this group will compare to practitioners and entrepreneurs, since the engagement of scientists in twitter communication varies considerably.

### 2.2 Hashtag analysis

Hashtags were searched in the text of individual tweets and grouped in different ways for different analyses. To investigate the average number of hashtags in tweets of different groups of users, hashtags were grouped per tweet and person. When comparing tweets and retweets, hashtags were grouped together regardless of user. To understand the frequency of contents represented by hashtags, we conducted a topic analysis using statistical topic modeling [43], which infers from the contents of hashtags the topic of the text. We employed a mixed-membership model to describe the data, for these methods describe a single text, a tweet, as a mixture of different topics. Analyzes were conducted using the package stm for R [44], which represents each document as a vector of proportions of words belonging to each topic. Hashtags occurring with a high frequency in a topic have a stronger impact on the topic than less frequent (or less specific) hashtags. Statistical topic modeling also allows the investigation of the effect of covariates on the distribution of document-topic proportions using logistic models [43]. We employed the covariate conversation (conversation length = 1 vs. >1) and date (2010–2022). Since data collection was stopped before 2022 finished, a spline was applied to adjust the estimation of the effect of date. Spectral initialization was employed to stabilize model estimation [44]. We estimated models with 10–30 different topics and examined the semantic coherence as well as the robustness of topic contents across the models with increasing numbers of topics. As suggested by Ref. [44], we also measured topic quality using the most frequent terms as well as the FREX index, which provides a combination of semantic coherence and exclusivity of words to topics. To ascertain the quality of each topic, one of the authors (GW) checked topic contents as well as text examples from the different topics.

### 2.3 Analysis of scientific credibility of neurofeedback research

Based on critical papers by Refs. [7,6] and the international consensus paper on quality criteria for neurofeedback studies [29], we analyzed tweets for the presence of terms representing the scientific credibility in neurofeedback research. These terms included “placebo”, “sham”, “control group” “double-blind”, “evidence”, “evidence based”, “questionable”, “transparency”, “checklist”, “Open Science”, “preregist*“r and “CRED-nf”.

### 2.4 Statistical analysis and summary of analytical approach

All statistical computations were calculated using the software R [45]. Frequency of tweets, analysis of profiles was performed with the package “sentiment.ai” [41] version 0.1.1. Wordclouds were constructed using the package “wordcloud”, version 2.6.

In the following, we provide a summary of the methodology of the present study with the aim to provide a reference on how to investigate public communication on topics that have quite different meanings to specific subcommunities and may involve the interaction between scientific experts, practitioners, equipment providers, and other users. We restricted the search to one single keyword (i.e., “neurofeedback”) as the technique has been widely known under this name world-wide over several decades [46].

We desired to understand the individual contributions of interested users to the communication on neurofeedback. For that, we determined a cut-off for interest based on the number of tweets published on neurofeedback by a specific user. The number of users reaching our (arbitrary) criterion of t ≽ 10 tweets was sufficient to identify about 1000 highly interested users. By doing that, we have an indication that for these users neurofeedback is a topic with enough relevance to impact their media identity and self-description. Indeed, after inspection of some short bios and definition of a few key-words, it was possible to employ a text embedment engine to calculate the similarities between the short bio descriptions and the lists of keywords assembled by us. In contrast to the requirements of hashtags, the aggregation of short bios required the use of text embedment engines sensitive to semantic properties of text.

Moreover, to approach the content of neurofeedback tweets we opted for a hashtag analysis. Inspection of the occurrence of hashtags revealed a large number of them being used in combination. So, we investigated their grouping using statistical text modeling STM [44]. This technique is an extension of Latent Dirichlet Analysis, that calculates the probabilities of co-occurrence of specific tokens and their specificity when computing topic analysis. Given the condensed nature of hashtags, text embedment engines would not be as useful to address the aggregation of hashtags and formation of topics as meaning insensitive STM. Moreover, STM also naturally assesses statistically the effect of covariates such as time on specific topics, so that we were able to assess which topics changed frequency in the time interval investigated.

Finally, we also aimed at determining the impact of specific scientific and non-scientific events on the communication about the clinical efficacy of neurofeedback. For that, we selected some specific events from the media involving neurofeedback as well as the publication date of influential papers on neurofeedback, some critical ones and another published with the aim of improving the level of clinical evidence on neurofeedback. We expected an event-related increase in the number of Twitter publications containing specific keywords connected to each one of these events. To evaluate the impact of each of these events, a baseline was established by determining the frequency of the specific contents in time intervals far from the time point of the respective events.

## 3 Results

### 3.1 Analysis of frequency of tweets

**Fig. 1:**
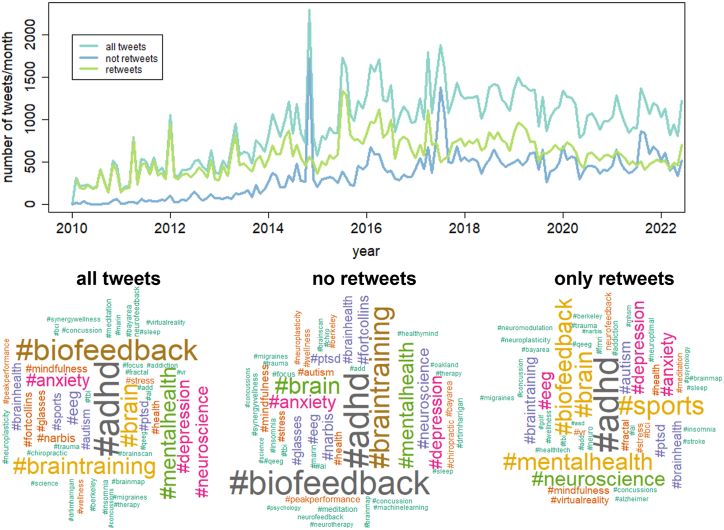
Above: Number of tweets/month containing the keyword “neurofeedback”. Below: Word clouds of the 50 most frequent hashtags in the categories all tweets, no retweets, and only retweets.

Searching for keyword “*neurofeedback*”, 138,819 tweets were obtained, which originated from 137,150 different conversations. From those, 985 conversations contained two tweets, only 248 more than 2 tweets, and merely 26 contained more than 5 tweets. Moreover, 38% of all tweets were retweeted at least once. The number of tweets on neurofeedback increased since 2010 and more or less stabilized since 2018 (Fig. 1). The community of users contains a total of 42,967 different profiles as of June 1st, 2022. Of them, 1221 profiles (2.8% of all profiles) have published more than 10 tweets on neurofeedback between 2010 and 2022, 160 profiles (0.4% of all profiles) more than 100 tweets and 6 profiles (0.01% of all profiles) have published more than 1000 tweets containing the keyword “*neurofeedback*” in the same time period. Fig. 1 shows the number of tweets/month separately for all tweets, tweets without and with at least one retweet, as well as Wordclouds containing their 50 most frequent hashtags.

**Fig. 2:**
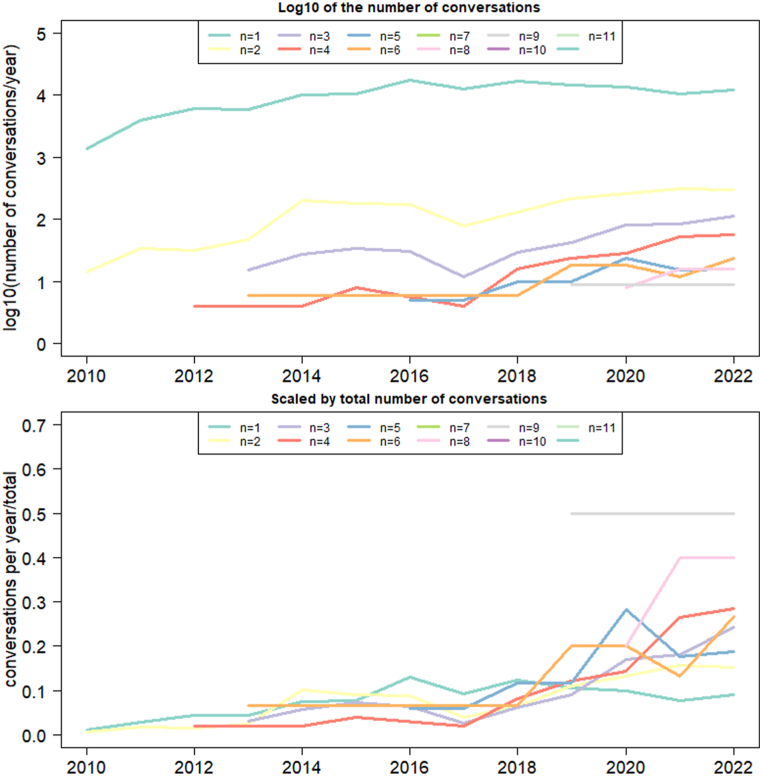
Frequency of conversations of different lengths per year. Top: Log10 of the number of conversations of each length. Bottom: Number of conversations scaled by the total number of conversations of each length in the interval between 2010 and 2022.

Analysis of the distribution of conversations beginning in the time interval between 2010 and 2022 reveals an increase in the number of conversations over the years. Particularly after 2017 the number of beginning conversations with more than 2 or 3 tweets increased in both absolute and relative terms (Fig. 2, top and bottom, respectively). When comparing the number of isolated tweets vs. tweets in a conversation between 2013-2016 and 2019–2022 reveals a decrease in the proportion of isolated tweets (98% vs. 97%, *χ*^*2*^ = 299.32, *df* = 1, *p* = 2.2*10^−16^) and a concomitant increase in the proportion of tweets in a conversation (2.0% vs 3.0%).

### 3.2 Analysis of profiles

**Fig. 3:**
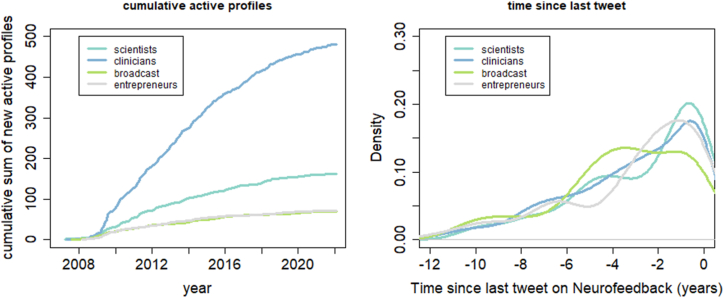
Left: Cumulative sum of the number of active twitter profiles, right: Time since the last tweet on neurofeedback.

Profile classification yielded cosine similarity values that did not differ between the four groups (i.e., scientists, broadcasts, entrepreneurs, clinicians; *MSE* = 0.001; *F* (3, 784) = 0.15, *p* = 9.3*10^−1^). This indicates that the classification accuracy by means of text embedment was similar across groups. The cumulative sum of active profiles of scientists, clinicians, broadcasts, and entrepreneurs is depicted in Fig. 3. The number of new active profiles is increasing in all four categories, but the rhythm of growth seems to decrease since at least 2015. Interestingly, the last tweet of profiles belonging to the different groups are contained approximately in the last 2 years, except in the case of entrepreneurs, who in large numbers published their last tweet on neurofeedback for the last time 4 or 5 years ago (i.e. in 2016 and 2017).

To validate the classification of profiles we asked a human unaware of the outcomes of the automatic classification to classify a sample of 100 profiles utilizing the same categories as available for sentiment. ai. Results were superior to chance for all categories. An agreement of 78% regarding the category “broadcast”, 62.5% in the category “entrepreneur”, 50% in the category “scientist” and 54% in the case of clinicians. Although the outcomes are far from perfect, it is important to consider how specific the concepts employed to classify the short bio were and can be considered as an acceptable classification of users of neurofeedback in different groups using their short bios.

The number of members of each group of profiles is depicted in Table 1 according to their total activity. The first number in each cell indicates the number of profiles with a good classification certainty and the second one indicates the total number of profiles regardless of the level of certainty. Among very active profiles 4 clinicians and 1 scientist were observed. Among profiles with a lower level of activity, all groups were observed. Interestingly, also among productive users mere 2%–3% of conversations were longer than a single tweet, as among other users.

**Table 1:** Publication activity among the different profile categories.

Level of activity	Scientist	Clinician	Broadcast	Entrepreneur
>1000 tweets	1/1	4/5	0/0	0/0
1000 > tweets ≥ 100	18/30	65/78	8/15	11/17
100 > tweets ≥ 10	142/204	412/527	61/140	59/112
conversations§	175/9827	617/34,956	98/3095	89/3704

Number of profiles with similarity <0.5/all profiles, § number of conversations with at least 2 tweets/total.

Table 2 shows the minimum, maximum and median of the number of followers and number of profiles followed by members of each one of the profile groups. Regarding numbers of followers and following, broadcast agencies showed the highest median values, while those shown by clinicians were the lowest ones.

**Table 2:** Descriptives of the number of followers and number of profiles being followed.

		Scientist	Clinicians	Broadcast	Entrepreneur
Followers	Min	3	0	74	1
	Max	219,366	268,449	167,680	281,561
	Median	898	376	1817	627
Following	Min	0	0	0	0
	Max	44,831	30,764	31,325	21,065
	Median	633	412	849	485

**Fig. 4:**
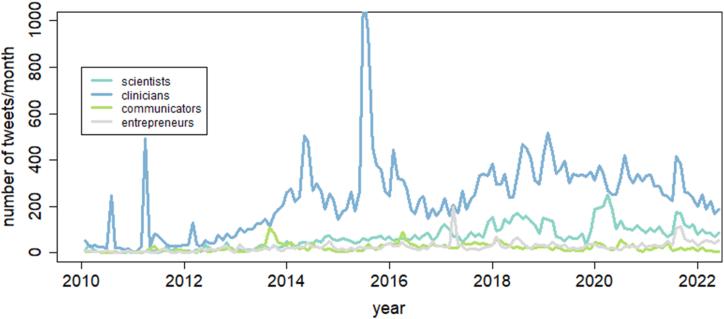
Average number of publications per month depicted separately for each group.

Fig. 4 shows the average number of tweets per month separately for each group.

The publication frequency among clinicians showed a large peak in 2015 and is decreasing since 2020. The publication frequency among scientists is increasing slowly since 2010. The number of publications among broadcasts remains constant while that of entrepreneurs increases slightly over the whole-time interval.

### 3.3 Analysis of hashtags

Hashtags were extracted from the text of each individual tweet containing them. When considering all tweets together, 13,418 different hashtags in 61,815 tweets were detected, meaning that 55% of all tweets did not contain any hashtag. The average number of hashtags per user was higher in retweets (μ = 1.56) than in tweets without a retweet (μ = 1.35). We then considered productive users and grouped the hashtags by individual, then compared user groups. Hashtags were aggregated per user and used to calculate group differences in a Poisson regression with the following model *grouped_hashtags ∼ user-group.* The intercept was larger than 0 (estimate = 1.41, std-error = 0.06, z-value = 20.95, p-value <2*10^−16^), indicating that in all groups a non-zero number of hashtags was used consistently by individuals. Regarding differences between groups of profiles, the non-significant results were obtained regardless of the function employed to group data: min, median, or max (all *p*-values >0.05).

Fig. 1 depicts wordclouds for the 50 most popular hashtags in each group. Font size indicates the frequency of hashtags. Unsurprisingly, “adhd” is the most frequent hashtag, followed by “biofeedback” and “braintraining” (Fig. 1). Interestingly, the hashtags “biofeedback” and “braintraining” are much more frequent among tweets without a retweet, while “sports”, “mental health”, and “neuroscience” are more typical of tweets with at least one retweet.

**Fig. 5:**
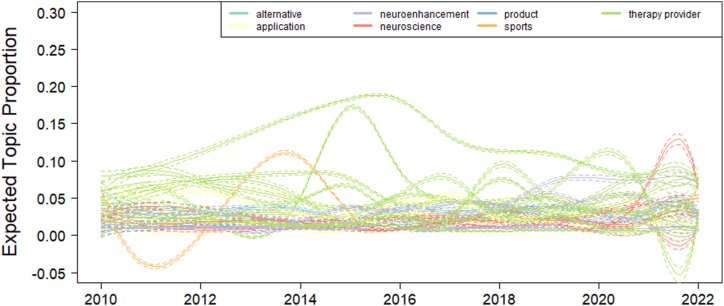
Topic prevalence plotted as a smooth function of time, holding rating at sample median, with 95% confidence intervals.

Topic analysis of hashtags revealed consistent outcomes across models with increasing numbers of topics. With content inspection of the topics, we observed strong semantic consistency across models. For this reason, outcomes of the model with 30 topics will be displayed. Topics can be further categorized in seven categories: Alternative practices, applications of neurofeedback, neuroenhancement, neuroscience, product, sports, and therapy provider. The features common to topics assigned to one of these groups were as follows: *Alternative practices* describe topics containing at least one alternative medicine practice. *Applications of neurofeedback* contained hashtags describing neurological or psychiatric populations or application fields of neurofeedback for health promotion, but no specific therapy provider. *Neuroenhancement* describes topics containing brain activation optimization, peak performance, etc. *Neuroscience* contained hashtags describing neuroscientific research. *Product* contained at least one hashtag describing one specific neurofeedback device brand. *Sports* described applications of neurofeedback to optimization of performance during sports. Finally, *therapy provider* contained topics with at least one hashtag referring to a specific therapy provider. Fig. 5 shows the topic prevalence as a function of time. Topics of the class therapy provider have on average the highest prevalence. Topics of the class neuroenhancement oscillate less in time and are growing in relevance. Sports showed a peak of relevance in 2014 and lost relevance after that. Neuroscience is growing in relevance since 2020.

Table 3 shows the top hashtags for each topic categorized according to their frequency only, as well as adjusted for exclusivity. As can be depicted from Table 3, the classification of topics into categories is approximate, since most topics also contain elements from more than a single category.

**Table 3:** Topic description by top hashtags.

Topic	Category	Effect of conversation	Topic contents
Topic 1 E = 3%	application	conversation	Highest Prob: brain, eeg, braintrain, neuroplast, brainmap, mind, brainscan FREX: neuroplast, brainmap, brainscan, healthymind, cfs, chronicfatigu, brainexercis
Topic 2 E = 0.2%	neuroscience	no conversation	Highest Prob: neurofeedback, ohbm, biorxiv, biorxivneursci, hy., ohbmpost, fearless FREX: ohbm, biorxiv, biorxivneursci, ohbmpost, fearless, sciencetakeslongerthancontract, protocol
Topic 3 E = 9%	therapy provider	no conversation	Highest Prob: braintrain, narbi, fortcollin, glass, fractal, brain, brainrenew FREX: glass, psychk, brainspot, narbi, brainrenew, skinonlin, skinonlineblog
Topic 4 E = 0.4%	neuroenhancement	indifferent	Highest Prob: trainyourbrain, constantcontact, retweet, brainpow, ploson, mindcontrol, mindhack FREX: constantcontact, retweet, ploson, mindcontrol, mindhack, assessmentandd, doganxieti
Topic 5 E = 3.3%	sports	conversation	Highest Prob: sport, bci, fmri, learn, exercis, devo, mental FREX: sport, devo, lifefit, openaccess, infograph, holobrain, magicl
Topic 6 E = 1.5%	alternative therapy	no conversation	Highest Prob: therapi, treatment, emot, heal, recoveri, medic, integr FREX: emot, heal, integr, acupunctur, tnc, hope, review
Topic 7 E = 0.5%	application	indifferent	Highest Prob: tinnitus, pediatr, lifestyl, focusband, equestrian, esport, scien FREX: tinnitus, pediatr, lifestyl, focusband, equestrian, esport, scien
Topic 8 E = 2.5%	neuroenhancement	no conversation	Highest Prob: qeeg, tech, healthtech, podcast, emotionalintellig, fit, digitalhealth FREX: qeeg, tech, healthtech, digitalhealth, healthcar, rtms, wearabletech
Topic 9 E = 0.9%	product	no conversation	Highest Prob: neuroptim, biohack, psychotherapi, ces, braintrain, neurohack, bipolardisord FREX: neuroptim, ces, neurohack, bipolardisord, rocketsci, denver, nic
Topic 10 E = 4.5%	application	no conversation	Highest Prob: autism, concuss, addict, tbi, migrain, add, chronicpain FREX: cte, bloodpressur, constip, digest, autismpar, obopportun, nhl
Topic 11 E = 4%	therapy provider	indifferent	Highest Prob: biofeedback, neurofeedback, train, dfw, southlak, hrv, olymp FREX: dfw, southlak, neurofeedbackcanhelp, advancedneurotherapi, adhdbrain, reactiontim, veteransday
Topic 12 E = 1.2%	application	conversation	Highest Prob: parent, thursdaythought, children, autist, famili, dyslexia, school FREX: parent, thursdaythought, famili, dyslexia, school, kidshealth, neurofeedba
Topic 13 E = 0.6%	therapy provider	indifferent	Highest Prob: braininjuri, soundcloud, rehabilit, free, blogtalkradio, gammabi, drakeinstitutecomplaint FREX: braininjuri, soundcloud, gammabi, eurekamag, tvomindweek, fredericton, neurvanahealth
Topic 14 E = 2.8%	application	indifferent	Highest Prob: health, well, alzheim, neuromodul, motiv, nutrit, braincor FREX: health, alzheim, amaz, ave, adopt, spiritu, gonstead
Topic 15 E = 1.8%	neuroscience	indifferent	Highest Prob: neurosci, asd, memori, epilepsi, cognit, neurotech, mindfulsci FREX: neu, neurotechnolog, biometr, mentalpow, mindabl, artificialintellig, san
Topic 16 E = 6%	therapy provider	indifferent	Highest Prob: adhd, neurotherapi, uniquemindcar, houston, adhdsymptom, mentalhealthclin, educ FREX: uniquemindcar, houston, adhdsymptom, mentalhealthclin, focusmadefun, addadhd, adultadhd
Topic 17 E = 2%	alternative	indifferent	Highest Prob: neuro, scienc, neurolog, neurosci, brain, cancer, game FREX: neuro, cancer, northcarolina, psych, adhdtreat, alternativemedicin, extivita
Topic 18 E = 0.8%	neuroscience	conversation	Highest Prob: machinelearn, neurofeedbacktherapi, iot, book, videogam, phd, dalla FREX: machinelearn, neurofeedbacktherapi, iot, phd, iiot, ecnp, neuroeth
Topic 19 E = 5%	therapy provider	conversation	Highest Prob: anxieti, depress, bayarea, berkeley, insomnia, marin, sleep FREX: anxieti, bayarea, berkeley, marin, oakland, albani, albanyca
Topic 20 E = 23%	therapy provider	no conversation	Highest Prob: neurofeedback, mobileeeg, webcours, oculus, lenstherapi, slt, tonbridgewel FREX: mobileeeg, oculus, lenstherapi, sarasota, somaticfunctionaltherapi, tonbridgewel, insight
Topic 21 E = 0.7%	neuroscience	indifferent	Highest Prob: psycholog, technolog, research, blog, bipolar, medicin, brainact FREX: psycholog, research, blog, bcia, neuromedit, articl, bfe
Topic 22 E = 3.5%	therapy provider	indifferent	Highest Prob: stress, virtualr, stroke, covid, job, thebraintrain, braintrainingcapetown FREX: virtualr, thebraintrain, braintrainingcapetown, changeyourlif, neurof, immun, canada
Topic 23 E = 3.2%	therapy provider	indifferent	Highest Prob: chiropract, drtimharrigan, synergywel, chiro, eurofeedback, tucson, mentalhealthservic FREX: drtimharrigan, synergywel, chiro, eurofeedback, tucson, innateintellig, nft
Topic 24 E = 2.4%	neuroenhancement	indifferent	Highest Prob: peakperform, brainwav, athlet, news, mentalfit, mef, mentaledg FREX: peakperform, mef, mentaledg, busi, trainwithus, video, success
Topic 25 E = 2.6%	neuroenhancement	conversation	Highest Prob: mind, medit, focus, perform, golf, calm, product FREX: product, neuroperforma, almostth, pga, tuesdaythought, muse, meditationpartoflif
Topic 26 E = 2%	application	no conversation	Highest Prob: ptsd, trauma, stemcel, molliisuit, smallstep, veteran, journey FREX: stemcel, molliisuit, smallstep, veteran, journey, isnr, brainpaint
Topic 27 E = 0.2%	neuroenhancement	no conversation	Highest Prob: bodybrainhealth, cbt, neurofeedback, chemobrain, mri, diet, hypnosi FREX: bodybrainhealth, chemobrain, mri, diet, hypnosi, neuroscientist, impuls
Topic 28 E = 3.5%	therapy provider	indifferent	Highest Prob: mentalhealth, neurosci, brainhealth, mentalhealthawar, brainhack, mentalhealthmatt, drtrishleigh FREX: brainhack, drtrishleigh, leighbrainandspin, gbt, braintrainin, mentalhea, intelligencecommun
Topic 29 E = 0.7%	therapy provider	no conversation	Highest Prob: parkinson, neurofeedbacktrain, mybraindr, neurotherapi, music, brainawarenessweek, eeger FREX: parkinson, brainawarenessweek, eegbiofeedback, eneuro, socialwork, parkinsonsdiseas, ptsdchat
Topic 30 E = 1%	neuroenhancement	indifferent	Highest Prob: neurofeedback, clearmind, brain, alzheimersdiseas, mendedmind, geteqsmart, improveperform FREX: neurofeedback, geteqsmart, fsm, cohen, overthink, bodybrainhealthi, portlandoregon

### 3.4 Scientific credibility of neurofeedback research

**Fig. 6:**
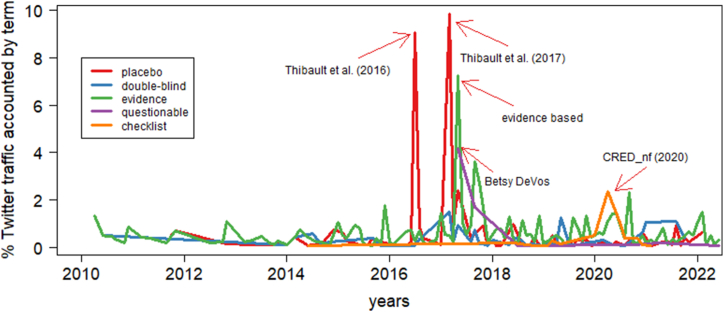
Proportion of whole neurofeedback-related twitter traffic accounted by specific terms related to scientific credibility of neurofeedback.

Some terms representing scientific credibility in neurofeedback research were defined according to recent scientific publications (e.g. “placebo”, “double-blind”, and “questionable”). Table 4 displays the total number of tweets per keyword, the number of original tweets, number of retweets, and the number of different authors. The keyword “placebo” showed peaks of activity that mobilized up to 10% of whole neurofeedback-related twitter traffic especially between 2016 and 2018, where critical papers of [[6], [7], [8], [24]] were published (Fig. 6). The key-words “double-blind” and “questionable” also showed an increase in frequency but of considerably smaller magnitude and at a later time. Post-hoc inspection of content revealed the trigger for tweets including the keyword “questionable” to be the Betsy DeVos event [22]. Interestingly, the keyword “evidence” contained mostly only advertisement and non-scientific references to use of neurofeedback and a few references to older neurofeedback studies [47,48]. The key-word “checklist” started to be mentioned in tweets in 2019, when the CRED-nf checklist was published as a preprint [30] and showed a peak in 2020, when the CRED-nf checklist was published in the scientific journal Brain [29].

**Table 4:** Characteristics of tweets related to scientific credibility.

Keyword	nr Tweets (% of tweets)	no retweets	sum retweets	nr authors
placebo, sham, control group	461 (0.33%)	230	1328	379
double-blind	160 (0.12%)	55	458	119
evidence	781 (0.56%)	385	2204	564
questionable, transparency	68 (0.05%)	46	279	67
checklist, open science, standard, preregistr*	66 (0.05%)	25	435	44
Total	1536 (1.11%)	741	4704	561^a^

a Number of unique profiles publishing to this topic.

To investigate the existence of a more permanent impact of the critical studies (i.e. mainly the publications by Robert Thibault and Amir Raz [[6], [7], [8], [24]]) on the neurofeedback-related twitter traffic, we compared the proportion of tweets including any of the keywords mentioned above in the time intervals January 2013–December 2015 vs. January 2019–December 2021. The selection of those two time intervals aimed at excluding the transient peak of interest observed in years 2016–2017 (see Fig. 6). The number of tweets containing these key-words doubled from 0.4% of the total Twitter traffic related to neurofeedback in 2013–2015 to 0.9% in 2019–2021 (*χ*^*2*^ = 66.4, *df* = 1, *p* = 3.7*10^−16^).

We also investigated the participation of productive users in publications regarding scientific credibility of neurofeedback. Among the profiles with up to 10 neurofeedback tweets, participation was below 1% (301/41,746 profiles), among the profiles with 11–100 neurofeedback tweets was 5% (55/1061 profiles), among the profiles with 101–1000 neurofeedback tweets was 15% (23/154 profiles), and 0% among profiles with more than 1000 (0/6 profiles) neurofeedback tweets. Tweets by users posting less than 10 times on the topic of neurofeedback account for the rest of the communication (89%).

The analysis of profiles with more than 10 neurofeedback tweets according to the categories scientist, clinician, broadcast and entrepreneur revealed a participation of 11% among scientists (17/164), 8% among clinicians (38/481), 9% among broadcasters (6/67) as well as 3% among entrepreneurs (2/73). No significant difference in group participation was detected (*χ*^*2*^ = 4.0, *df* = 3, *p* = 2.7*10^−1^).

## 4 Discussion

In the present study, we investigated the public discourse on Twitter around the topic neurofeedback. The number of tweets including the term “neurofeedback” increased steadily from 2010 to 2018 and seemed to stabilize since then. The majority of tweets was generated by a few profiles [49] and only few conversations with two or more tweets were observed. Regarding both peer-to-peer interactions as well as scientific communication, the reduced number and length of conversations indicates that the communication flow in the community is mainly unidirectional and non-interactive. However, the number and length of conversations has been increasing in the last few years. Analysis of the short bio of productive users revealed four user categories, scientists, clinicians, entrepreneurs and broadcast, which show specific preferences in communication. Topic analysis revealed that therapy providers concentrate prevalent topics, followed by application fields, neuroenhancement, and topics related to neuroscience. The relative prevalence of therapy provider topics seems to decrease, applications and neuroenhancement topics remain unchanged, and neuroscience seems to become more popular in the last few years. Finally, specific reactions to events related to the scientific credibility of neurofeedback were observed as well as changes in the communication patterns. In the following, these results will be discussed in more depth.

### 4.1 General features of communication on neurofeedback

The number of tweets per year increased in the first half of the last decade and seems to remain stable since 2018. Until 2016 the communication was almost exclusively unilateral and showed only a few interactions; conversations with more than one tweet are rare but are becoming more common in recent years. Interestingly, the low number of conversations with more than one tweet indicate that Twitter has not been employed frequently for active peer-to-peer interaction [28] or search for advice [18]. Although these more intensive forms of peer-to-peer interaction around the topic neurofeedback seem to be the exception, some alternative forms of social recognition can still be given in the form of likes or retweets [1], which were however not analyzed in the present study. Still, our results differ from those reviewed by Ref. [11], which are more typical of health information seeking. Neurofeedback does not seem to be a typical topic in health information seeking, but contains a blend of motivations that involve not only the search for health information [11] but also neuroenhancement [50], lifestyle and wellness [51]. In the section hashtag analysis, we will come back to this finding. In summary, conversations remain a peripheral phenomenon in the public communication on the topic neurofeedback.

### 4.2 Profile analysis

One of the aims of the present study was to provide a tentative characterization of user profiles communicating on the topic neurofeedback. Using the criteria of 10 tweets on neurofeedback, less than 1% of the users were classified as productive users. We employed their Twitter short bios as a container of information on user's characteristics [20]. Twitter profile descriptions are considered an expression of aspects of their social identity [31] and can help to understand users' interests on, and motivations for interaction with Twitter contents [52]. Among productive users, different subgroups of productive users were identified: Scientists, broadcasts, entrepreneurs, and clinicians. This classification of profiles is useful, for Twitter is a platform for scientific communication as well as for the communication between researchers, providers, and clients.

The majority of productive users tweeting about neurofeedback belong to the group of clinicians. The second largest group is the group of scientists and the number of profiles belonging to broadcasts and entrepreneurs is of comparable size. The semantic classification of short bios using a semantic latent analysis engine produced acceptable results which passed our validity criteria but did not involve any validation of scientific or therapeutic activity connected to each profile. Therefore, our analyses do not allow us to distinguish between individuals with strong interest in science or therapy and those with specific training to perform these activities professionally. The profiles of broadcasts showed the highest median number of followers and simultaneously are following the largest number of profiles. Moreover, publications by broadcasts also generated with the highest probability a conversation with at least two tweets. These findings are consistent with their main purpose in the promotion of scientific communication. The lowest median number of followers was observed among clinicians. We also interpret these results as consistent with the limited geographical radius of action typical of clinicians in comparison to the other groups. However, a few clinicians have a wider reach since the maximum number of followers is the second highest of all groups. This also fits to the finding by the Pew Research Center that a small number of twitter profiles have a wide reach.

The number of new users belonging to the different subgroups is changing at different paces. While the number of clinicians interested in neurofeedback shows rather stagnation, the number of broadcasts, entrepreneurs and scientists is increasing in the last years. Moreover, the different subgroups also show specific publication behavior. While clinicians showed a peak of activity before 2016 and after that a monthly activity never superior to 2014, broadcasts, entrepreneurs and scientists, although much less numerous than clinicians, are publishing more frequently in the last few years. These results are suggestive regarding changes in the composition of the network communicating on the topic of neurofeedback.

The increase in the twitter activity of the group of scientists may lead to stronger communication of the state-of-the-art evidence on neurofeedback and help correct the divergence [50] between scientific literature and promises of neurofeedback services on the internet [[6], [7], [8], [24]]. As pointed out by Ref. [35], the majority of followers of scientists are other scientists and only those profiles with more than 1000 followers also reach a broader audience consisting of research and educational organizations, media, members of the public with no stated association with science, and decision-makers. If the estimates by Ref. [35] are valid also for the scientific communication on neurofeedback, the mass of scientific communication necessary to fill the gap between the commercial exploitation of neurofeedback and an evidence-based form of treatment [8], has not yet been reached, but is also not far away (Table 2).

Moreover, the communication on neurofeedback does not resemble the pattern identified by Ref. [17] when analyzing providers of treatments lacking scientific evidence. To the contrary, a stable community of promoters and supporters was identified in the present study. However, the kind of treatment investigated by Ref. [17] lack any form of scientific scrutiny, while the neural mechanisms and clinical efficacy of neurofeedback have been investigated and published [53], even if not as often as desirable [6]. Even if the problems with neurofeedback's reputation are more subtle than those investigated in the alternative medicine scene by Ref. [17], pure evidence-based treatment is still far from reality in the practice of neurofeedback. One possible reason for the inertia is the so-called “neuroenchantment” [25], a lack of critical thought towards the possibilities and achievements of neurotechnology [25]. did not have to put much effort into making between 63% and 75% of the college students believe that it was possible to read their brain activity and thoughts with a large hair dryer. The lack of critical reasoning observed by Ref. [25] is prevalent among laymen as well as experts and can be observed in the form of neuromyths [54]. The fascination exerted by easily recordable brain activity, such as EEG neurofeedback devices, feeds strong expectations and even engenders new normative frameworks for the self in both users and practitioners alike [26]. The stronger participation of scientists in the twitter communication on neurofeedback may indicate a change of course. In the next sections, this discussion will be extended by considering the influence of hashtags and specific events on the communication about neurofeedback.

### 4.3 Hashtag analysis

We addressed the question of which contents are related with neurofeedback in Twitter interactions by means of a hashtag analysis, for these, index and categorize tweets’ contents [33] and can be seen as distilled versions of conceptual frameworks and viewpoints [34]. Tweets on neurofeedback contain a large number of unique hashtags. The use of more hashtags was associated with higher probability of retweeting neurofeedback contents. This is in line with previous findings relating the use of hashtags with the popularity of tweets [1,12]. The most frequently used hashtag was #adhd, which is the shortcut for attention-deficit/hyperactivity disorder. This is not surprising since the use of neurofeedback to reduce ADHD symptoms is one of the most common clinical applications of neurofeedback. Additionally, the majority of research studies investigating the effects of neurofeedback training focussed on ADHD [55,56,57,3]. Biofeedback is also frequently mentioned, since both are conceptually and clinically related [58]. Beside ADHD, different clinical applications of neurofeedback (e.g., #anxiety, #depression) are also frequently mentioned. As pointed out above, however, the small number of conversations suggests that the pattern of interaction typical of search for advice and emotional support was not observed in the data set.

When comparing the frequency of hashtags between tweets and retweets, non-clinical applications of neurofeedback such as #sports, #golf, or #virtualreality are more prevalent in retweets. Topic analysis revealed the existence of a few classes of topics. By far the most frequent topic can be characterized as the self-presentation of specific therapy providers. Other frequent topics are application fields and neuroenhancement. Other forms of treatment mentioned are muscle relaxation (e.g. Exopulse Mollii Suit), biofeedback, chiropractors as well as controversial interventions such as eye movement desensitization and reprocessing and brainspotting. Application fields have contents similar to those by therapy providers, but among the most important hashtags no specific references to practitioners or their institutions can be detected. The topics of neuroenhancement comprise all the aspects of lifestyle and wellness related to self-optimization by means of neurofeedback or a combination of neurofeedback and other high-tech techniques such as computer games and virtual reality. As pointed out by Ref. [37], hashtags define communities and communication spaces for users to represent themselves and identify with other users. The topics identified above reveal that the self-presentation of specific therapy providers concentrates the largest part of hashtag use, followed by application fields, neuroenhancement and, more recently, neuroscience.

Analysis of the time courses of specific topics revealed the most prevalent topics to be filled by therapy practitioners, followed by application fields and neuroenhancement. While the prevalence of therapy practitioners decreased in the last few years, a recent increase in the prevalence of neuroscience topics has been observed. Application fields and neuroenhancement topics did not show considerable changes of prevalence in the time interval investigated in the present study. In summary, the public discourse on neurofeedback is characterized by a map of application fields, which contains the targets of neurofeedback interventions (i.e. specific psychiatric and neurological disorders), as well as in many cases the reference to concrete practitioners. It also contains the promises of benefit, which range from the outcomes of treatment of specific body and mental disorders to the achievement of performance optimization through neuroenhancement.

### 4.4 Scientific credibility of neurofeedback research

Finally, we investigated the reactions generated by scientific and critical findings on neurofeedback-related Twitter traffic. When analyzing the public response to specific events regarding the scientific credibility of neurofeedback, we observed an increase in the frequency of some important key-words on twitter. Peaks of activity were related to the publication of specific critical studies [7,6], the Betsy deVos event as well as the CRED_nf checklist (2020). While the Betsy deVos event can be discounted because of its rather political [22] than scientific character, the other peaks of activation are connected to a change in the public discourse on neurofeedback. The use of vocabulary referring to the scientific credibility of neurofeedback more than doubled from 2013 to 2015 to 2019–2021. Although it represents only a modest fraction of the neurofeedback-related twitter traffic, this increase in awareness goes beyond the mere peak of interest on this topic registered in 2016 and 2017. An increase in the awareness of placebo, double-blind controlled studies as well as open science practices is a highly desirable outcome of scientific communication on neurofeedback.

However, caution in the interpretation of the consequences of this increase of awareness is required. A closer look at the keyword “evidence” reveals that its peak follows that of the publication of [6] study by only a few months. Inspection of the content of the tweets containing the keyword “evidence” disclosed not new scientific reports on evidence-based neurofeedback but rather advertisement of neurofeedback services and devices containing this keyword. As pointed out by Ref. [19], language adaptation is a measurable reaction to stress and threat. In the case of the keyword “evidence”, the increase in its usage reveals an adaptation of vocabulary made relevant by critical publications *without* the generation of new scientific evidence. These results are in line with the findings by Ref. [50] regarding a considerable divergence between scientific evidence on the efficacy of neurofeedback and the marketing of products and services in websites of neurofeedback providers. The extent to which genuine changes in scientific practice and standards for services involving neurofeedback drive the vocabulary of public discourse on neurofeedback will require more studies.

[36] investigated the quality of interactions and public engagement in five different scientific fields and observed that most communication activities remain undigested, with no sign of debate or collective reflection. Following the critical publications since 2016 a higher degree of interaction can be observed in the communication on neurofeedback. First, the number and length of conversations on neurofeedback are increasing and differ significantly from the pattern observed between 2013 and 2016. Moreover, the participation of scientists also increased in the same period. Although still incipient, the trend observed the communication on neurofeedback is promising. As a contribution to intensify the interactive character of scientific communication on Twitter, the authors created a twitter channel @NFResearchGraz in which we will publish relevant scientific new findings on neurofeedback not only by linking to the publications but also by discussing it in text. We plan to contribute one tweet per week containing text about an actual topic in neurofeedback research, which is meant to motivate more dialogue and exchange [35,36] on Twitter. Moreover, we will post a twitter with a link to every new paper containing the CRED-nf checklist in its reference list. This practice is meant to serve as a milestone in scientific communication on twitter and can help to evaluate the impact of Open Science and good practices in scientific communication about neurofeedback as well as an impulse for its spread among non-scientific users.

## 5 Limitations

As the very first study of public communication on the topic of neurofeedback conducted using Twitter data, our study has several limitations. Our rationale was to keep the conceptualization as straightforward as possible, for the relative impact of different motives to communicate about neurofeedback have not been charted hitherto. In our view, the results we obtained corroborate this decision, for we spotted through bio classification and topic analysis very diverse motives for communication on neurofeedback. Accordingly, in future studies there are many different directions in which the communication on neurofeedback can be tracked and investigated in more detail: neurofeedback as an example of (i) neuroenhancement and neurohacking [59], (ii) or as an example of modern entertainment or wellness, (iii) or as an example of therapy in specific disorders such as anxiety, depression and ADHD, (iv) or of technique to achieve peak performance in sports, or (v) the connection with related techniques such as biofeedback. The keywords connected to each of these examples vary wildly and involve separate communities with very different communication motives and habits. For this reason, we decided to focus on the keyword neurofeedback alone in this very first step, understand its fundamental characteristics and proceed from there in a specific direction.

For instance, in the case of biofeedback, we do not expect the same strong association with neuroenhancement as neurofeedback but rather a more typical clinical communication character, for many of the grandiloquent promises of neurofeedback towards neuroenhancement have never been made towards biofeedback. For all these reasons we refrained from extending the query to other keywords in this very first study but have plans to do that in follow-up studies.

Another limitation refers to the validation of short bio classification with the help of human classification of a subsample of profiles. Although in all cases classification was at least twice as high as expected by chance, a substantial discrepancy between text embedment engine and the human classifier was present. Obviously, this procedure of short bio classification only works for a proportion of users and deserves further validation steps in future studies.

## 6 Conclusions

Communication on neurofeedback is complex and driven by many different motives and expectations at once. It includes many other reasons to employ neurofeedback beyond scientific investigation and treatment, such as entertainment, wellness, and the desire to optimize performance and enhance oneself. It cannot be reduced to interactions typically seen in scientific communication, alternative medicine, or in health domains, although it certainly contains elements of all of them. As pointed out by Ref. [60], the more complex the topic, the more prevalent the epistemological tensions, which may favor malpractice and render scientific investigation less important in science-to-public communication. Communication on neurofeedback As pointed out by Ref. [26], some brain -related technologies are perceived as an instrument to allow a particular kind of intimacy with oneself, which is otherwise impossible without the help of brain measurements. Neurofeedback has been identified as a strong example of such technologies. As pointed out by Ref. [61], technologies have the power to self-reproduce and evolve in non-predictable ways. Accordingly, neurofeedback inspires developers, scientists, users, and providers to expand its field of application. As a powerful motive for fantasies of empowerment for experts and laypeople alike, neurofeedback is a seductive technique still largely immune to scientific scrutiny. This has implications for both industry and scientific communication.

Implications for the industry: although communication on neurofeedback is clearly distinct from that of alternative medicine, it still drifts in a gray zone in which higher standards for responsible communication would be desirable. In line with the study by Ref. [50], we observed a divergence between the scientific literature on neurofeedback and the marketing of neurofeedback services to the general public, which can be considered misleading to the general public. We consider the so-called neuroenchantment [25] an important force underlying the communication on brain-training related topics, of which neurofeedback is only one example. Neuroenchantment describes an optimistic appraisal of brain-reading and brain-stimulation systems and the non-critical acceptance of their promises. Neuroenchantment is not restricted to laypersons but also affects individuals with sufficient background in neuroscience. It is not uncommon among neurofeedback system developers to target not only the health branch but also the entertainment and optimization branches and even direct-to-consumer services [16]. As pointed out by Ref. [62], the risk/benefit ratio is different for treating diseases versus enhancing functions and requires better communication to non-clinical clients.

Implications for the academic community: The last few years saw an increase in the relative participation of the scientific community in the communication about neurofeedback. This has consequences for both scientific and science-to-public communication. A more intensive Twitter communication on neurofeedback reveals an increase in the scientific interest on the topic and efforts to produce, communicate and discuss scientific advances in this domain. One central part of it is the topic of clinical efficacy, which in several instances of neurofeedback application is still far from solid [63,64,65]. As we observed, scientific communication is characterized more by advertisement of new studies than by in-depth discussions of them [36]. More discussion on the merits and flaws of specific neurofeedback training protocols represents one of the most important tasks of science communicators regarding the topic of neurofeedback. These efforts also should have an impact on the kind of advertisement of applications visible through Twitter and bring them closer to the state-of-art in clinical research. As we showed here, the impact of scientific communication on Twitter conversations about neurofeedback is still modest but growing, so that only time will tell whether the current efforts will succeed. One open question remains though, whether the scientific community and other user communities will interact more intensively in the future and exchange on the topics of clinical efficacy of neurofeedback applications and thereby overcome the negative effects of neuroenchantment. Our findings indicate the existence of a large potential of Twitter to be used more effectively in scientific communication on neurofeedback as it is already employed in public health and educational settings [66,67,68,69]. Twitter can be seen as a useful tool to build awareness for good practices in neurofeedback research. This will require a more consistent engagement of researchers.

## Author contribution statement

Guilherme Wood: Conceived and designed the experiments; Analyzed and interpreted the data; Wrote the paper.

Finn Buchrieser; Analyzed and interpreted the data; Wrote the paper.

Silvia Erika Kober; Conceived and designed the experiments; Analyzed and interpreted the data; Wrote the paper.

## Data availability statement

The authors do not have permission to share data.

## Additional information

No additional information is available for this paper.

## Declaration of competing interest

The authors declare that they have no known competing financial interests or personal relationships that could have appeared to influence the work reported in this paper.

## References

[1] Lahuerta-Otero E., Cordero-Gutiérrez R., De la Prieta-Pintado F. (2018). Retweet or like? That is the question. Online Inf. Rev..

[2] Arns M., Batail J.-M., Bioulac S., Congedo M., Daudet C., Drapier D., Fovet T., Jardri R., Le-Van-Quyen M., Lotte F., Mehler D., Micoulaud-Franchi J.-A., Purper-Ouakil D., Vialatte (2017). Neurofeedback: one of today's techniques in psychiatry?. L'encephale.

[3] Enriquez-Geppert S., Smit D., Pimenta M.G., Arns M. (2019). Neurofeedback as a treatment intervention in ADHD: current evidence and practice. Curr. Psychiatr. Rep..

[4] Sterman M.B., Egner T. (2006). Foundation and practice of neurofeedback for the treatment of epilepsy. Appl. Psychophysiol. Biofeedback.

[5] Marzbani H., Marateb H., Mansourian M. (2016). Methodological note: neurofeedback: A comprehensive review on system design, methodology and clinical applications. Basic and Clinical Neuroscience Journal.

[6] Thibault R.T., Raz A. (2017). The psychology of neurofeedback: clinical intervention even if applied placebo. Am. Psychol..

[7] Thibault R.T., Lifshitz M., Raz A. (2016). The self-regulating brain and neurofeedback: experimental science and clinical promise. Cortex; a Journal Devoted to the Study of the Nervous System and Behavior.

[8] Thibault R.T., Lifshitz M., Raz A. (2018). The climate of neurofeedback: scientific rigour and the perils of ideology. Brain : J. Neurol..

[9] Wood G., Kober S.E. (2018). EEG neurofeedback is under strong control of psychosocial factors. Appl. Psychophysiol. Biofeedback.

[10] Naslund J.A., Aschbrenner K.A., Marsch L.A., Bartels S.J. (2016). The future of mental health care: peer-to-peer support and social media. Epidemiol. Psychiatr. Sci..

[11] Zhao Y., Zhang J. (2017). Consumer health information seeking in social media: a literature review. Health Inf. Libr. J..

[12] Su L.Y.-F., Scheufele D.A., Bell L., Brossard D., Xenos M.A. (2017). Information-sharing and community-building: exploring the use of twitter in science public relations. Sci. Commun..

[13] Toraman C., Şahinuç F., Yilmaz E.H., Akkaya I.B. (2022). Understanding social engagements: a comparative analysis of user and text features in Twitter. Social Network Analysis and Mining.

[14] Fischer E., Reuber A.R. (2014). Online entrepreneurial communication: mitigating uncertainty and increasing differentiation via Twitter. J. Bus. Ventur..

[15] Suh B., Hong L., Pirolli P., Chi E.H. (2010, August). 2010 IEEE Second International Conference on Social Computing.

[16] Wexler A. (2018). Who uses direct-to-consumer brain stimulation products, and why? A study of home users of tDCS devices. Journal of Cognitive Enhancement.

[17] Lavorgna A., Carr L. (2021). Tweets and quacks: network and content analyses of providers of non-science-based anticancer treatments and their supporters on twitter. Sage Open.

[18] Antheunis M.L., Tates K., Nieboer T.E. (2013). Patients' and health professionals' use of social media in health care: motives, barriers and expectations. Patient Educ. Counsel..

[19] Garcia D., Rimé B. (2019). Collective emotions and social resilience in the digital traces after a terrorist attack. Psychol. Sci..

[20] Pennacchiotti M., Popescu A.-M. (2011). A machine learning approach to twitter user classification. Proceedings of the International AAAI Conference on Web and Social Media.

[21] Popescu A.-M., Pennacchiotti M., Huang X.J. (2010). CIKM'10: Proceedings of the 19th International Conference on Information & Knowledge Management and Co-located Workshops.

[22] Boser U. (2017). Betsy DeVos has invested millions in this ‘brain training’ company. So I checked it out. At a clinic in Florida, I found that Neurocore seems to be promising more than it can deliver. Washingtonpost.

[23] Fink S., Eder S., Goldstein M. (2017).

[24] Thibault R.T., Lifshitz M., Birbaumer N., Raz A. (2015). Neurofeedback, self-regulation, and brain imaging: clinical science and fad in the service of mental disorders. Psychother. Psychosom..

[25] Ali S.S., Lifshitz M., Raz A. (2014). Empirical neuroenchantment: from reading minds to thinking critically. Front. Hum. Neurosci..

[26] Littlefield M.M. (2018).

[27] Millington B. (2012). Use it or lose it: ageing and the politics of brain training. Leisure Stud..

[28] Neubauer A.C., Wood G. (2022). Intelligenzsteigerung durch Neuroenhancement?. Psychol. Rundsch..

[29] Ros T., Enriquez-Geppert S., Zotev V., Young K.D., Wood G., Whitfield-Gabrieli S., Wan F., Vuilleumier P., Vialatte F., van de Ville D., Todder D., Surmeli T., Sulzer J.S., Strehl U., Sterman M.B., Steiner N.J., Sorger B., Soekadar S.R., Sitaram R., Thibault R.T. (2020). Consensus on the reporting and experimental design of clinical and cognitive-behavioural neurofeedback studies (CRED-nf checklist). Brain : J. Neurol..

[30] Ros T., Enriquez-Geppert S., Zotev V., Young K., Wood G., Whitfield-Gabrieli S., Wan F., Vialatte F., van de Ville D., Todder D., Surmeli T., Sulzer J., Strehl U., Sterman B., Steiner N., Sorger B., Soekadar S., Sitaram R., Sherlin L., Thibault R.T. (2019).

[31] Priante, A., Hiemstra, D., van den Broek, T., Saeed, A., Ehrenhard, M., & Need, A. #WhoAmI in 160 Characters? Classifying Social Identities Based on Twitter Profile Descriptions. In D. Bamman, A. S. Doğruöz, J. Eisenstein, D. Hovy, D. Jurgens, B. O'Connor, A. Oh, O. Tsur, & S. Volkova (Eds.), Proceedings of the First Workshop on NLP and Computational Social Science (pp. 55–65). Association for Computational Linguistics 10.18653/v1/W16-5608.

[32] Ghosh S., Ekbal A., Bhattacharyya P. (2022). What does your bio say? Inferring twitter users' depression status from multimodal profile information using deep learning. IEEE Transactions on Computational Social Systems.

[33] Bonilla Y., Rosa J. (2015). #Ferguson: digital protest, hashtag ethnography, and the racial politics of social media in the United States. Am. Ethnol..

[34] Xu S., Zhou A. (2020). Hashtag homophily in twitter network: examining a controversial cause-related marketing campaign. Comput. Hum. Behav..

[35] Côté I.M., Darling E.S. (2018). Scientists on Twitter: preaching to the choir or singing from the rooftops?. FACETS.

[36] Didegah F., Mejlgaard N., Sørensen M.P. (2018). Investigating the quality of interactions and public engagement around scientific papers on Twitter. Journal of Informetrics.

[37] Jackson S.J., Foucault Welles B. (2015). Hijacking #myNYPD: social media dissent and networked counterpublics. J. Commun..

[38] Araujo R.F. (2020). Communities of attention networks: introducing qualitative and conversational perspectives for altmetrics. Scientometrics.

[39] Barrie C., Ho J. (2021). academictwitteR: an R package to access the Twitter Academic Research Product Track v2 API endpoint. J. Open Source Softw..

[40] Kearney M. (2019). rtweet: collecting and analyzing Twitter data. J. Open Source Softw..

[41] Wiseman B., Nydick S., Wisner T. (2022). https://CRAN.R-project.org/package=sentiment.ai.

[42] Rahutomo F., Kitasuka T., Kitasuka, Aritsugi M. (2012). Semantic cosine similarity. In the 7th International Student Conference on Advanced Science and Technology ICAST.

[43] Roberts M.E., Stewart B.M., Tingley D., Lucas C., Leder-Luis J., Gadarian S.K., Rand D.G. (2014). Structural topic models for open‐ended survey responses. Am. J. Polit. Sci..

[44] Roberts M.E., Stewart B.M., Tingley D. (2019). Stm : an R package for structural topic models. J. Stat. Software.

[45] R Core Team (2022). https://www.R-project.org/.

[46] Rubí M.C.M. (2007). Neurofeedback around the world. J. Neurother..

[47] Hodgson K., Hutchinson A.D., Denson L. (2014). Nonpharmacological treatments for ADHD: a meta-analytic review. J. Atten. Disord..

[48] Ros T., Munneke M.A., Ruge D., Gruzelier J.H., Rothwell J.C. (2010). Endogenous control of waking brain rhythms induces neuroplasticity in humans. Eur. J. Neurosci..

[49] Wojick S., Hughes A. (2019).

[50] Wexler A., Nagappan A., Kopyto D., Choi R. (2020). Neuroenhancement for sale: assessing the website claims of neurofeedback providers in the United States. Journal of Cognitive Enhancement.

[51] Pilař L., Kvasničková Stanislavská L., Kvasnička R. (2021). Healthy food on the twitter social network: vegan, homemade, and organic food. Int. J. Environ. Res. Publ. Health.

[52] Abuhassan M., Anwar T., Fuller-Tyszkiewicz M., Jarman H.K., Shatte A., Liu C., Sukunesan S. (2023). Classification of Twitter users with eating disorder engagement: learning from the biographies. Comput. Hum. Behav..

[53] Sitaram R., Ros T., Stoeckel L., Haller S., Scharnowski F., Lewis-Peacock J., Sulzer J. (2017). Closed-loop brain training: the science of neurofeedback. Nat. Rev. Neurosci..

[54] van Elk M. (2019). Socio-cognitive biases are associated to belief in neuromyths and cognitive enhancement: a pre-registered study. Pers. Indiv. Differ..

[55] Arns M., Clark C.R., Trullinger M., deBeus R., Mack M., Aniftos M. (2020). Neurofeedback and attention-deficit/hyperactivity-disorder (ADHD) in children: rating the evidence and proposed guidelines. Appl. Psychophysiol. Biofeedback.

[56] Arns M., Heinrich H., Strehl U. (2014). Evaluation of neurofeedback in ADHD: the long and winding road. Biol. Psychol..

[57] Cortese S., Ferrin M., Brandeis D., Holtmann M., Aggensteiner P., Daley D., Santosh P., Simonoff E., Stevenson J., Stringaris A., Sonuga-Barke E.J.S. (2016). Neurofeedback for attention-deficit/hyperactivity disorder: meta-analysis of clinical and neuropsychological outcomes from randomized controlled trials. J. Am. Acad. Child Adolesc. Psychiatr..

[58] Gaume A., Vialatte A., Mora-Sánchez A., Ramdani C., Vialatte F.B. (2016). A psychoengineering paradigm for the neurocognitive mechanisms of biofeedback and neurofeedback. Neurosci. Biobehav. Rev..

[59] Ienca M., Scheibner J. (2020). Developments in Neuroethics and Bioethics.

[60] Sauerborn E., Sökefeld N., Neckel S. (2022). Paradoxes of mindfulness: the specious promises of a contemporary practice. Socio. Rev..

[61] Ellul J. (2021).

[62] Wurzman R., Hamilton R.H., Pascual-Leone A., Fox M.D. (2016). An open letter concerning do-it-yourself users of transcranial direct current stimulation. Ann. Neurol..

[63] Dudek E., Dodell-Feder D. (2020). The efficacy of real-time functional magnetic resonance imaging neurofeedback for psychiatric illness: a meta-analysis of brain and behavioral outcomes. Neurosci. Biobehav. Rev..

[64] Ming-Qiang X., Xiao-Hui H., Ba-Gen L., Jingwen L., Min H. (2018). The effect of neurofeedback training for sport performance in athletes: a meta-analysis. Psychol. Sport Exerc..

[65] Patel K., Sutherland H., Henshaw J. (2020). Effects of neurofeedback in the management of chronic pain: a systematic review and meta-analysis of clinical trials. Eur. J. Pain.

[66] Edo-Osagie O., La Iglesia B. de, Lake I., Edeghere O. (2020). A scoping review of the use of Twitter for public health research. Comput. Biol. Med..

[67] Ginger Pinholster, Ham Becky (2013). Science communication requires time, trust, and twitter. Science.

[68] Kaseda E. (2020). https://www.apa.org/science/about/psa/2020/05/academic-twitter.

[69] Malik A., Heyman-Schrum C., Johri A. (2019). Use of Twitter across educational settings: a review of the literature. International Journal of Educational Technology in Higher Education.

